# Consumption of caffeinated beverages and kidney function decline in an elderly Mediterranean population with metabolic syndrome

**DOI:** 10.1038/s41598-021-88028-7

**Published:** 2021-04-22

**Authors:** Andrés Díaz-López, Indira Paz-Graniel, Verónica Ruiz, Estefanía Toledo, Nerea Becerra-Tomás, Dolores Corella, Olga Castañer, J. Alfredo Martínez, Ángel M. Alonso-Gómez, Julia Wärnberg, Jesús Vioque, Dora Romaguera, José López-Miranda, Ramon Estruch, Francisco J. Tinahones, José Lapetra, Luís Serra-Majem, Aurora Bueno-Cavanillas, Josep A. Tur, Vicente Martín Sánchez, Xavier Pintó, Miguel Delgado-Rodríguez, Pilar Matía-Martín, Josep Vidal, Clotilde Vázquez, Lidia Daimiel, Tania Fernandez Villa, Emilio Ros, Sonia Eguaras, Nancy Babio, Jose V. Sorlí, Albert Goday, Itziar Abete, Lucas Tojal Sierra, Francisco Javier Barón-López, Laura Torres-Collado, Marga Morey, Antonio Garcia-Rios, Rosa Casas, María Rosa Bernal-López, José Manuel Santos-Lozano, Adela Navarro, Jose I. Gonzalez, María Dolores Zomeño, Maria Angeles Zulet, Jessica Vaquero Luna, Raul Ramallal, Montse Fitó, Jordi Salas-Salvadó

**Affiliations:** 1 Serra Hunter Fellow, Universitat Rovira i Virgili (URV), 43201 Reus, Spain; 2 Unitat de Nutrició, Departament de Bioquímica i Biotecnologia, Rovira i Virgili University, 43201 Reus, Spain; 3 Institut d’Investigació Sanitària Pere Virgili (IISPV), 43201 Reus, Spain; 4 Centro de Investigación Biomédica en Red Fisiopatología de la Obesidad y la Nutrición (CIBEROBN), Institute of Health Carlos III, 28029 Madrid, Spain; 5 Nutrition Unit, University Hospital of Sant Joan de Reus, 43201 Reus, Spain; 6 Department of Preventive Medicine and Public Health, IdiSNA, University of Navarra, 31008 Pamplona, Spain; 7 Department of Preventive Medicine, University of Valencia, Av. de Blasco Ibáñez, 15, 46010 Valencia, Spain; 8 Cardiovascular Risk and Nutrition Research Group (CARIN), Hospital del Mar Research Institute (IMIM), 08003 Barcelona, Spain; 9 Department of Nutrition, Food Science and Physiology, IdiSNA, University of Navarra, 31008 Pamplona, Spain; 10 Nutritional Genomics and Epigenomics Group, IMDEA Food, CEI UAM + CSIC, 28049 Madrid, Spain; 11 Bioaraba Health Research Institute, Osakidetza Basque Health Service, Araba University Hospital, University of the Basque Country UPV/EHU, Vitoria-Gasteiz, Spain; 12 Department of Nursing, Institute of Biomedical Research in Malaga (IBIMA), University of Málaga, 29071 Malaga, Spain; 13 ISABIAL-FISABIO, Miguel Hernandez University, 46020 Alicante, Spain; 14 CIBER de Epidemiología y Salud Pública (CIBERESP), Instituto de Salud Carlos III, 28029 Madrid, Spain; 15 Health Research Institute of the Balearic Islands (IdISBa), 07120 Palma de Mallorca, Spain; 16 Department of Internal Medicine, Maimonides Biomedical Research Institute of Cordoba (IMIBIC), Reina Sofia University Hospital, University of Cordoba, 14004 Córdoba, Spain; 17 Department of Internal Medicine, Institutd’Investigacions Biomèdiques August Pi Sunyer (IDIBAPS), Hospital Clinic, University of Barcelona, 08036 Barcelona, Spain; 18 Department of Endocrinology, Instituto de Investigación Biomédica de Málaga (IBIMA), Virgen de la Victoria Hospital, University of Málaga, 29010 Malaga, Spain; 19 Research Unit, Department of Family Medicine, Distrito Sanitario Atención Primaria Sevilla, 41013 Seville, Spain; 20 Research Institute of Biomedical and Health Sciences (IUIBS), Preventive Medicine Service, Centro Hospitalario Universitario Insular Materno Infantil (CHUIMI), Canarian Health Service, University of Las Palmas de Gran Canaria, 35016 Las Palmas, Spain; 21 Department of Preventive Medicine, University of Granada, 18071 Granada, Spain; 22 Research Group on Community Nutrition and Oxidative Stress, University of Balearic Islands, 07122 Palma de Mallorca, Spain; 23 Institute of Biomedicine (IBIOMED), University of León, 24071 León, Spain; 24 Lipids and Vascular Risk Unit, Internal Medicine, Hospital Universitario de Bellvitge, Hospitalet de Llobregat, 08907 Barcelona, Spain; 25 Division of Preventive Medicine, Faculty of Medicine, University of Jaén, 23071 Jaén, Spain; 26 Department of Endocrinology and Nutrition, Instituto de Investigación Sanitaria Hospital Clínico San Carlos (IdISSC), 27040 Madrid, Spain; 27 Department of Endocrinology, IDIBAPS, Hospital Clínic, University of Barcelona, 08036 Barcelona, Spain; 28 CIBER Diabetes y Enfermedades Metabólicas (CIBERDEM), Instituto de Salud Carlos III (ISCIII), 28029 Madrid, Spain; 29 Department of Endocrinology and Nutrition, Hospital Fundación Jimenez Díaz, Instituto de Investigaciones Biomédicas IISFJD, University Autonoma, 28040 Madrid, Spain; 30 Lipid Clinic, Department of Endocrinology and Nutrition, Institut d’Investigacions Biomèdiques August Pi Sunyer (IDIBAPS), Hospital Clínic, 08036 Barcelona, Spain; 31 Department of Public Health, Instituto de Investigación Biomédica de Málaga (IBIMA), University of Málaga, Malaga, Spain; 32 Human Nutrition Unit, Faculty of Medicine and Health Sciences, Universitat Rovira i Virgili, C/Sant Llorenç 21, 43201 Reus, Spain

**Keywords:** Nephrology, Risk factors

## Abstract

It remains unclear whether caffeinated beverages could have deleterious renal effects in elderly population with underlying comorbid conditions. We investigated the associations between coffee, tea, or caffeine intake and 1-year changes in glomerular filtration rate (eGFR) in a large Spanish cohort of overweight/obese elderly with metabolic syndrome (MetS). This prospective analysis includes 5851 overweight/obese adults (55–75 years) with MetS from the PREDIMED-Plus study. We assessed coffee, tea, and caffeine consumption from a validated food-frequency questionnaire and creatinine-based eGFR using the Chronic Kidney Disease Epidemiology Collaboration equation. Multivariate-adjusted regression models were applied to test associations between baseline coffee, tea, or caffeine intake and 1-year eGFR changes. Caffeinated coffee (> 2 cups/day) and tea (at least 1 cup/day) drinkers had 0.88 and 0.93 mL/min/1.73 m^2^ greater eGFR decrease respectively, compared to those with less than 1 cup/day of coffee consumption or non-tea drinkers. Furthermore, caffeinated coffee consumption of > 2 cups/day was associated with 1.19-fold increased risk of rapid eGFR decline > 3 mL/min/1.73 m^2^ (95% CI 1.01–1.41). Similarly, individuals in the highest (median, 51.2 mg/day) tertile of caffeine intake had a 0.87 mL/min/1.73 m^2^ greater eGFR decrease. Decaffeinated coffee was not associated with eGFR changes. In conclusion, higher consumption of caffeinated coffee, tea, and caffeine was associated with a greater 1-year eGFR decline in overweight/obese adults with MetS.

## Introduction

Chronic kidney disease (CKD) poses a major public health challenge due to its detrimental impact on quality of life, increased morbidity and premature death, specifically in old people^1^. This heterogeneous condition is characterized by a decline of glomerular filtration rate (eGFR) and/or proteinuria^2^, which is accelerated when obesity or related cardiovascular risk factors are present^3^. Therefore, preserving renal function is essential to ensure the well-being and reduce adverse health outcomes in elders.

Many dietary components play a role in preserving renal function and preventing/delaying CKD progression^4,5^, such as coffee and tea^6,7^, two caffeine-containing beverages that are widely consumed worldwide. Coffee and tea are rich in bioactive substances, such as phenolic compounds and minerals with antioxidant and anti-inflammatory properties, which can improve blood pressure, oxidative stress, dyslipidemia, and insulin resistance or hyperglycemia^8,9^, well-established risk factors for renal insufficiency. There is evidence pointing to a protective effect of coffee and tea against type 2 diabetes, hypertension, and cardiovascular disease, conditions in which chronic inflammation plays a critical pathogenic role^10–12^. Nonetheless, there are potential side effects from caffeine contained in these beverages, even at moderate amounts, which may influence CKD-related cardiovascular outcomes and are of concern for the general population and especially for susceptible or at-risk individuals^13^.

The results of epidemiological studies on coffee and tea consumption and kidney function have been inconsistent^14–23^. Two recent meta-analyses, summarizing 7 cross-sectional studies on apparently healthy middle-aged adults, most from Asian countries, have concluded that coffee consumption is not associated with eGFR or CKD risk^6,7^. Regarding prospective studies; some studies reported a lower incidence of CKD^21,24^ or end-stage renal disease^19^ with greater coffee consumption, while others failed to demonstrate this association^22^. For tea consumption, the few studies examining its association with eGFR or CKD risk reported null findings^15,19,20,22^. Several limitations of these studies need to be addressed; (1) most previous studies are cross-sectional rather than longitudinal studies, (2) they only reported information on total coffee consumption and not its subtypes (caffeinated and decaffeinated coffee), (3) stratified analyses were often not reported, and (4) they were performed in general healthy adults, and not in vulnerable elderly individuals. In addition, the impact of coffee or tea on eGFR could be different among countries with different lifestyle and tea and coffee-drinking habits. Consequently, it is unclear whether caffeinated beverages have deleterious effects on kidney function in elderly population with underlying comorbid conditions. Therefore, the purpose of this study was to address the associations of caffeinated and decaffeinated coffee, tea, and caffeine consumption with 1-year changes in eGFR in a large Spanish cohort of overweight/obese older individuals with metabolic syndrome (MetS).

## Methods

### Study design and participants

The present data was analysed using an observational prospective design conducted within the frame of the PREDIMED-PLUS study, which included 6874 older adults enrolled between 2013 and 2016 by 23 Spanish centers working in collaboration with 208 National Health System primary care clinics. Briefly, PREDIMED-Plus is an ongoing, 6-year, multicenter, parallel randomized clinical trial (RCT) evaluating the long-term effect of a weight-loss intervention based on an energy-reduced Mediterranean diet (MedDiet), physical activity promotion and behavioral support (intervention), in comparison with usual-care recommending an energy-unreduced MedDiet (control), on primary cardiovascular prevention. Eligible participants were overweight/obese (BMI 27–40 kg/m^2^) men and women aged 55–75 years harboring the MetS^25^, but free of cardiovascular disease at enrollment. More specific details of the study cohort and inclusion/exclusion criteria have been reported^26,27^ and the protocol is available at http://predimedplus.com/. Local Ethics Committee approved the study protocol and all participants signed a written informed consent.

For the current study, participants who did not complete the semiquantitative food-frequency questionnaire (SFFQ) at baseline (n = 53) or those whose total energy intake was outside pre-defined limits (women < 500 and > 3500 kcal/day, and men < 800 and > 4000 kcal/day) (n = 188) were excluded. We also excluded subjects who died (n = 11) or were lost to follow-up (n = 16) within first year of follow-up, and who had missing data on eGFR at baseline or at the 1-year assessment. The remaining 5851 participants comprised the final sample.

### Assessment of coffee, tea, and caffeine

The exposures of interest were baseline consumption of coffee (caffeinated and decaffeinated coffee), tea, and total caffeine intake. Trained dietitians collected participant information about dietary intake through face-to-face interviews using a Spanish version of the validated 143-item SFFQ^28^. Participants reported their average frequency of consumption over the preceding year for a specified serving size of each item. The nine possible answers ranged from “never or less than once per month” to “six or more per day”, which were transformed into grams or milliliters per day using the standard portion size of each food and beverage. Two and one items on the FFQ were specifically related to coffee consumption (one for caffeinated coffee and one for decaffeinated coffee) and tea consumption, respectively. The standard serving size of 1 cup of coffee or tea was assigned as 50 mL in the questionnaire. Total coffee consumption was considered as the sum of caffeinated and decaffeinated coffee. Spanish food composition tables were used to estimate daily energy and nutrient intakes^29^.

We modeled three categories of coffee consumption (0 to < 1cup/day, 1–2 cups/day, and > 2 cups/day) and tea consumption (0 cup/day, < 1 cup/day, and ≥ 1 cups/day) with the lowest category as the referent group. Total caffeine intake was computed from the SFFQ using the caffeine contained in caffeinated coffee (400 mg/L), decaffeinated coffee (10.7 mg/L), tea (100 mg/L), regular sodas (79.2 mg/L), artificially sweetened soda (128 mg/L) and chocolate (180 mg/kg). Reference values from the European Food Safety Authority^30^ were used to calculate caffeine intake. For data analyses, caffeine intake (mg/day) was categorized into tertiles. We did not collect any information on the types of tea, soda, and chocolate.

### Assessment of the outcome and covariates

The main outcome of interest for the present study was 1-year changes in kidney function, assessed by changes in eGFR (1-year data minus baseline data) estimated indirectly from serum creatinine (SCr) using the Chronic Kidney Disease Epidemiology Collaboration equation for Caucasian individuals^31^. SCr levels were determined by the enzymatic creatinine assay method (coefficient of variation < 4.0%). The secondary outcome was a rapid decline of kidney function, defined as 1-year eGFR decline rate > 3 mL/min/1.73 m^2^. This threshold represents a magnitude of change that is 3 times the decline rate imposed by natural ageing.

At baseline, trained staff collected participant’s information through questionnaires regarding sociodemographic characteristics (sex, age, education, and employment status), lifestyles factors (smoking, physical activity^32,33^), history of illnesses and medication use. Additionally, we assessed adherence to the MedDiet using a 17-item questionnaire, adapted from a previously validated questionnaire^34^. This tool was used to evaluate compliance with the intervention and as a key element to guide the motivational interviews during the follow-up. Compliance with each of the 17 items relating to characteristic food habits was scored with 1 or 0 points, when the criterion was met or not met, respectively. Therefore, the total MedDiet score range was 0–17, with 0 meaning no adherence and 17 meaning maximum adherence to MedDiet. Weight, height and waist circumference were determined in duplicate by trained personnel following a pre-established protocol. BMI was calculated by dividing the weight (kg) by the height squared (m^2^). Blood pressure was measured at rest in triplicate by an automated digital device (Omron-HEM297705C). At baseline and 1-year, blood samples were collected after an overnight fast, and routine biochemical analyses including fasting glucose, lipid profile, and SCr was performed.

### Statistical analyses

All analyses were performed by STATA software, v.15.0 (StataCorp LP, Tx. USA) using the PREDIMED-Plus database updated in March 2019.

Descriptive data are presented as mean ± SD for quantitative variables or percentages for qualitative variables. Baseline characteristics of the study population across categories of total coffee and tea consumption were compared using ANOVA and chi-square tests, as appropriate.

Multivariate linear regression models were applied to test associations between categories (lowest category as reference) of coffee, tea, and caffeine intake and changes in eGFR at 1 year. For these associations, coffee, tea, (both for each cup/day increase) and caffeine (for each 50 mg/day increase) were also analyzed as continuous variables. The results are presented as β-coefficients and 95% confidence intervals (CIs). Furthermore, ORs were calculated for the association between categories of coffee and tea consumption and rapid eGFR decline (1-year changes in eGFR > 3 mL/min/1.73 m^2^). Three models with additional adjustment for potential confounders were fitted: model 1, adjusted for sex (men/women), age (years, continuous), and baseline eGFR (mL/min/1.73 m^2^, continuous); model 2, additionally adjusted for BMI (kg/m^2^, continuous), smoking (never, current or former smoker), education (primary, secondary education or academic/graduate), physical activity (MET-min/day, continuous), type 2 diabetes (yes/no), dyslipidemia (yes/no), hypertension (yes/no), center (four categories by number of recruited participants: < 200, 200–250, 250–300, or ≥ 300), treatment group (intervention/control), MedDiet score (17-points, continuous), and total energy intake (kcal/day, quartiles); to minimize confounding by other dietary factors, a third model (model 3) was additionally adjusted for dietary intakes of total protein, saturated fat, alcohol, fiber, sodium, magnesium, potassium (each categorized at energy-adjusted quartiles by using the residual method^35^), 1-year changes in systolic blood pressure (mmHg, continuous), and 1-year changes body weight (kg, continuous). Model 3 was further adjusted (cup/day, continuous) for coffee (for tea), tea (for each coffee), caffeinated coffee (for decaffeinated coffee), or decaffeinated coffee (for caffeinated coffee). All models were conducted with robust variance estimators to account for intra-cluster correlations, considering as clusters the members of the same household (n = 354 couples). Tests for linear trends across categories were conducted by modeling the median value for coffee, tea, or caffeine intake categories as continuous variables.

Furthermore, stratified analyses were conducted by intervention group (intervention/control), sex, age (≤ 65/ > 65 years), baseline eGFR (≥ 90 mL/min/1.73 m^2^, 60–89 mL/min/1.73 m^2^, < 60 mL/min/1.73 m^2^), obesity (yes/no), type 2 diabetes (yes/no), hypertension (yes/no), dyslipidemia (yes/no), smoking (non-smoker/smoker or ex-smoker), and MedDiet score (≤ 10/ > 10-points [P75]); The potential effect modification for eGFR by these stratification variables was explored by likelihood-ratio tests comparing the models with and without cross-product interaction terms, after adjusting for model 3.

Finally, we performed a sensitivity analysis, in which we repeated the primary analysis but using percentage of eGFR change ((1-year eGFR − baseline eGFR)/(baseline eGFR) * 100) instead of eGFR change, adjusting for baseline eGFR. Statistical significance was set at *p* < 0.05.

### Ethics approval and consent to participate

The study protocol and procedures were approved according to the ethical standards of the Declaration of Helsinki by the Institutional Review Boards (IRBs) of all the participating institutions: CEI Provincial de Málaga, CEI de los Hospitales Universitarios Virgen Macarena y Virgen del Rocío, CEI de la Universidad de Navarra, CEI de las Illes Balears, CEIC del Hospital Clínic de Barcelona, CEIC del Parc de Salut Mar, CEIC del Hospital Universitari Sant Joan de Reus, CEI del Hospital Universitario San Cecilio, CEIC de la Fundación Jiménez Díaz, CEIC Euskadi, CEI en Humanos de la Universidad de Valencia, CEIC del Hospital Universitario de Gran Canaria Doctor Negrín, CEIC del Hospital Universitario de Bellvitge, CEIC de IMDEA Alimentación, CEIC del Hospital Clínico San Carlos, CEI Provincial de Málaga, CCEIBA de la Investigación Biomédica de Andalucía, CEIC del Hospital General Universitario de Elche, Comité de Ética del Hospital Universitario Reina Sofía and CEIC de León. All participants provided informed written consent.

## Results

At baseline, the mean age of participants was 65 years, the mean BMI 32.5 kg/m^2^, and 48% were women. The mean baseline eGFR was 84.2 mL/min/1.73 m^2^ and mean change at 1 year was − 0.96 mL/min/1.73 m^2^. Mean daily consumption of caffeinated coffee, decaffeinated coffee, and tea were 42 mL/day (0.8 cup/day), 36 mL/day (0.7 cup/day), and 11 mL/day (0.2 cup/day) respectively. The mean intake of caffeine was 24.8 ± 25.7 mg/day. Table 1 shows baseline characteristics of study population by categories of coffee and tea consumption. Overall, 91.7% of participants were daily coffee consumers, and 36.4% drank > 2 cups of coffee/day. In contrast, only 32.5% were tea consumers and 11.3% drank at least 1 cup of tea/day. Compared with participants who consumed coffee less than daily (< 1 cup/day), those who consumed > 2 cups/day were more likely to be men, whereas tea consumers were more frequently women. Individuals who drank > 2 cups/day of coffee were younger and had higher BMI and baseline eGFR. They were more likely to have obesity, diabetes, and dyslipidemia and more frequently used oral hypoglycemic drugs, smoked, had higher waist circumference, fasting glucose and educational levels, and lower MedDiet adherence. For tea consumption, the associations were mostly in the opposite direction, except for education. High coffee consumption was also associated with a high intake of saturated fat, protein, sodium, magnesium, and potassium and with low fiber intake. Similar trends were observed for tea, except for saturated fat, fiber and alcohol intake, which showed an opposite pattern.

**Table 1:** General characteristics of the studied population according to categories of total coffee and tea consumption.

	Coffee consumption, cups/day			Tea consumption, cups/day		
	0 to < 1cup/day	1–2 cups/day	> 2 cups/day	0 cup/day	< 1cup/day	≥ 1 cups/day
	n = 1091	n = 2631	n = 2129	n = 3948	n = 1243	n = 660
Caffeinated coffee, mL/day	5.4 ± 11.3	28.4 ± 24.8	76.9 ± 71.5	42.4 ± 55.7	43.1 ± 52.2	35.1 ± 47.6
Decaffeinated coffee, mL/day	6.3 ± 10.3	28.6 ± 24.4	59.6 ± 66.5	37.5 ± 48.9	32.8 ± 45.8	30.4 ± 44.3
Tea, mL/day	16.5 ± 40.1	10.7 ± 25.9	9.3 ± 26.0	0.0	12.8 ± 10.5	75.9 ± 49.3
Caffeine, mg/day	11.5 ± 17.9	18.9 ± 15.7	38.9 ± 31.3	23.9 ± 25.9	25.2 ± 24.5	28.8 ± 23.8
Age, years	65.4 ± 4.9	65.3 ± 4.7	64.4 ± 5.0	65.1 ± 4.9	64.8 ± 4.7	64.9 ± 4.7
Women, %	47.8	52.9	41.8	42.9	56.9	60.9
BMI, kg/m^2^	32.3 ± 3.5	32.5 ± 3.4	32.7 ± 3.4	32.5 ± 3.4	32.5 ± 3.4	32.4 ± 3.4
Waist circumference, cm	106.7 ± 9.7	107.0 ± 0.2	108.6 ± 9.4	107.9 ± 9.6	106.9 ± 9.5	106.3 ± 10.1
Systolic blood pressure, mmHg	140.7 ± 17.7	139.1 ± 17.0	139.3 ± 16.3	140.2 ± 17.1	138.3 ± 16.5	137.2 ± 16.2
Diastolic blood pressure, mmHg	81.4 ± 10.1	80.6 ± 9.9	80.8 ± 9.7	81.1 ± 9.9	80.4 ± 9.6	79.8 ± 9.4
Plasma fasting glucose, mg/dL	111.9 ± 27.3	112.5 ± 27.9	115.4 ± 30.8	114.2 ± 29.8	111.9 ± 27.6	110.9 ± 25.9
Plasma HD-c, mg/dL	47.6 ± 11.8	48.8 ± 11.8	47.4 ± 11.7	47.5 ± 11.6	48.6 ± 11.4	50.3 ± 13.3
Plasma triglycerides, mg/dL	149.6 ± 75.6	150.7 ± 74.4	152.8 ± 78.0	151.7 ± 75.3	151.3 ± 79.3	148.6 ± 73.7
eGFR, mL/min/1.73 m^2^	83.8 ± 14.5	83.5 ± 13.9	85.19 ± 13.7	84.1 ± 14.1	84.5 ± 13.4	84.3 ± 14.5
**Comorbidities, %**						
Obesity (BMI ≥ 30 kg/m^2^)	69.9	72.1	75.9	73.5	73.4	72.6
Central obesity	94.0	96.4	96.7*	95.7	96.8	96.2
Type 2 diabetes^a^	27.3	30.1	32.8	31.7	30.2	24.7
Hypercholesterolemia	66.5	69.3	71.0	68.4	69.1	70.9
Hypertension	82.2	84.3	83.2	84.9	81.7	78.6
Low HDL-c	45.2	42.0	42.6	42.3	43.6	43.9
Hypertriglyceridemia	55.4	55.9	54.8	55.8	54.1	55.8
Hyperglycemia	75.7	74.2	76.7	75.8	75.2	73.3
CKD (eGFR < 60 mL/min/1.73 m^2^)	7.8	6.4	6.3	6.8	5.7	7.3
**Medication use, %**						
Lipid-lowering drugs	48.7	50.8	52.4	51.1	51.9	48.5
Oral blood glucose-lowering drugs	22.7	26.2	27.9	27.3	26.2	19.2
Insulin treatment	3.7	4.3	4.5	4.4	3.9	3.9
Antihypertensive drugs	77.6	78.2	78.1	79.2	76.3	74.8
ARBs	39.3	36.3	38.6	38.6	36.4	34.9
ACEis	31.6	31.9	30.9	32.3	29.5	30.6
**Education level, %**						
Primary education	48.6	52.2	46.1	51.3	45.4	44.7
Secondary education	30.9	28.2	29.4	29.1	29.6	28.5
Academic or graduate	20.4	19.6	24.5	18.6	25.0	26.8
**Smoking status, % (n)**						
Never smoked	49.5	48.5	36.4	42.7	48.9	45.1
Former smoker	41.5	41.0	46.4	43.6	40.5	44.7
Current smoker	9.0	10.5	17.1	13.7	10.5	10.1
Leisure time physical activity, METS. min. /week	375.8 ± 332.1	364.2 ± 338.6	350.6 ± 334.1	361.7 ± 347.5	356.6 ± 306.1	368.6 ± 317.9
MedDiet score (17-points)	8.9 ± 2.6	8.5 ± 2.7	8.2 ± 2.6	8.3 ± 2.6	8.6 ± 2.7	9.1 ± 2.7
**Nutrient intake/day**						
Energy, kcal	2310 ± 556	2325 ± 525	2455 ± 564	2377 ± 546	2363 ± 554	2339 ± 558
Saturated fat, g	26.0 ± 5.2	26.1 ± 5.1	26.7 ± 5.9	26.2 ± 5.2	26.8 ± 5.6	26.0 ± 5.9
Total protein, g	95.5 ± 14.1	97.5 ± 14.2	98.9 ± 15.5	96.8 ± 14.8	99.1 ± 14.2	99.7 ± 14.7
Fiber, g	26.9 ± 7.8	26.7 ± 7.9	25.0 ± 7.9	24.4 ± 7.8	27.0 ± 7.7	28.9 ± 8.4
Sodium, mg	2391 ± 509	2441 ± 530	2438 ± 542	2428 ± 544	2434 ± 498	2434 ± 515
Magnesium, mg	410.2 ± 75.8	421.3 ± 79.2	424.5 ± 84.4	413.3 ± 79.4	429.3 ± 78.1	446.3 ± 86.1
Potassium, mg	4245 ± 829	4481 ± 840	4537 ± 898	4408 ± 850	4561 ± 832	4724 ± 931
Alcohol, g	10.8 ± 14.2	11.0 ± 3.5	11.6 ± 15.1	12.2 ± 15.3	8.9 ± 11.4	9.3 ± 11.8

Values are means (± SD) and percentages for continuous and categorical variables, respectively.

*BMI* body mass index, *SBP* systolic blood pressure, *DBP* diastolic blood pressure, *HDL-c* high-density lipoprotein-cholesterol, *ARBs* angiotensin-type 2 receptor blockers, *ACEis* angiotensin converting enzyme inhibitors, *eGFR* estimated glomerular filtration rate, *MedDiet* Mediterranean diet.

^a^Current diabetes was defined as previous diagnosis of diabetes, glycated haemoglobin (HbA1c) ≥ 6.5%, use of antidiabetic medication, or having fasting glucose > 126 mg/dL in both the screening visit and baseline visit.

^b^*P* value for comparisons between categories of coffee consumption was tested by ANOVA or chi^2^, as appropriated.

The standard serving size of 1 cup was defined as 50 mL in the questionnaire.

Total coffee consumption was not significantly associated with eGFR changes: β: − 0.51; 95% CI − 1.17, 0.14 mL/min/1.73 m^2^ for > 2 cups/day compared with none to < 1 cup/day (*p*-trend = 0.07) in the fully adjusted model 3 (Table 2). Regarding coffee subtypes, caffeinated coffee consumption was associated with a 1-year eGFR decline in the model adjusted for age, sex, and baseline eGFR, with a significant β value of − 1.00 (95% CI − 1.52, − 0.45) mL/min/1.73 m^2^ for participants drinking > 2 cups/day compared with those in the lowest category (*p*-trend = 0.001). This association was not substantially affected after adjustment for other potential confounders in model 3, with an eGFR change of − 0.88 (95% CI − 1.52, − 0.23) mL/min/1.73 m^2^ lower in participants drinking > 2 cups/day compared to those with none to < 1 cup/day (p-trend = 0.008). When caffeinated coffee was analyzed as continuous, eGFR decreased by − 0.17 (95% CI − 0.40, 0.05) mL/min/1.73 m^2^ per 1 cup/day increment in the fully adjusted model, however, this result was not significant (*p* = 0.15). Conversely, the frequency of decaffeinated coffee consumption was not associated with changes in eGFR (Table 2).

**Table 2:** Crude and multivariate linear regression models of 1-year changes in eGFR according to categories of coffee and tea consumption at baseline.

	Coffee consumption, cups/day			*P* for trend	Per cup/day increase
	0 to < 1cup/day	1–2 cups/day	> 2 cups/day		
Total coffee, n (frequency)	1091	2631	2129		
eGFR change, mL/min/1.73 m^2^	− 0.75 (− 0.32, − 0.19)	− 0.73 (− 1.09, − 0.37)	− 1.36 (− 1.73, − 0.99)		
** versus 0 to < 1cup/day**					
Model 1	Ref	− 0.08 (− 0.70, 0.55)	− 0.52 (− 1.15, 0.11)	0.05	− 0.09 (− 0.29, 0.10)
Model 2	Ref	0.05 (− 0.57, 0.68)	− 0.31 (− 0.95, 0.32)	0.18	− 0.03 (− 0.22, 0.17)
Model 3	Ref	− 0.11 (− 0.74, 0.52)	− 0.51 (− 1.17, 0.14)	0.07	− 0.05 (− 0.26, 0.15)
Caffeinated coffee, n (frequency)	3169	1479	1203		
eGFR change, mL/min/1.73 m^2^	− 0.78 (− 1.11, − 0.46)	− 0.84 (− 1.30, − 0.37)	− 1.59 (− 2.07, − 1.10)		
** versus 0 to < 1cup/day**					
Model 1	Ref	− 0.33 (− 0.86, 0.21)	− 1.00 (− 1.56, − 0.45)^a^	0.001	− 0.26 (− 0.45, − 0.07)^b^
Model 2	Ref	− 0.30 (− 0.83, 0.23)	− 0.93 (− 1.49, − 0.37)^a^	0.001	− 0.23 (− 0.42, − 0.03)^b^
Model 3	Ref	− 0.23 (− 0.81, 0.36)	− 0.88 (− 1.52, − 0.23)^a^	0.008	− 0.17 (− 0.40, 0.05)
Decaffeinated coffee, n (frequency)	3381	1522	948		
eGFR change, mL/min/1.73 m^2^	− 1.04 (− 1.34, − 0.74)	− 0.70 (− 1.18, − 0.22)	− 1.10 (− 1.65, − 0.54)		
** versus 0 to < 1cup/day**					
Model 1	Ref	0.48 (− 0.05, 1.02)	0.40 (− 0.20, 1.02)	0.11	0.21 (− 0.01, 0.43)
Model 2	Ref	0.52 (− 0.02, 1.05)	0.53 (− 0.07, 1.13)	0.05	0.24 (− 0.02, 0.47)
Model 3	Ref	0.33 (− 0.27, 0.94)	0.36 (− 0.39, 1.10)	0.31	0.19 (− 0.09, 0.47)

	Tea consumption, cups/day			*P* for trend	Per cup/day increase
	0 cup/day	< 1cup/day	≥ 1 cups/day		
Tea, n (frequency)	3948	1243	660		
eGFR change, mL/min/1.73 m^2^	− 0.85 (− 1.13, − 0.55)	− 1.07 (− 1.57, − 0.58)	− 1.44 (− 2.13, − 0.74)		
**versus 0 cup/day**					
Model 1	Ref	− 0.19 (− 0.73, 0.36)	− 0.61 (− 1.33, 0.11)	0.10	− 0.38 (− 0.75, − 0.01)^b^
Model 2	Ref	− 0.31 (− 0.85, 0.24)	− 0.89 (− 1.62, − 0.16)^a^	0.02	− 0.54 (− 0.92, − 0.17)^b^
Model 3	Ref	− 0.29 (− 0.84, 0.26)	− 0.93 (− 1.67, − 0.18)^a^	0.02	− 0.55 (− 0.93, − 0.16)^b^

Values are means (95% CI) unless otherwise indicated.

*eGFR* estimated glomerular filtration rate, *Ref.* referent value. *P* value for trend based on multivariate linear regression treating the median values of each category as an ordinal variable.

Model 1: adjusted for sex (men or women), age (years, continuous), and baseline eGFR (mL/min/1.73 m^2^, continuous).

Model 2: additionally adjusted for BMI (kg/m^2^, continuous), smoking (never, current, or former smoker), educational level (primary, secondary education or academic/graduate), physical activity (MET-min/day, continuous), type 2 diabetes prevalence (yes or no), dyslipidemia (yes or no), hypertension (yes or no), center (four categories by number of recruited participants: < 200, 200–250, 250–300, or ≥ 300), intervention group (intervention or control group), MedDiet score (17-points, continuous) and total energy intake (kcal/day, quartiles).

Model 3: additional adjusted for dietary intakes of total protein, saturated fat, alcohol, fiber, sodium, magnesium, potassium (each categorized by energy-adjusted quartiles), 1-year changes in systolic blood pressure (mm Hg, continuous) and 1-year changes body weight (kg, continuous). Model 3 was further adjusted (cup/day, continuous) for coffee (for tea analysis), tea (for each coffee analysis), caffeinated coffee (for decaffeinated coffee analysis), or decaffeinated coffee (for caffeinated coffee analysis). All analyses were conducted with robust estimates of the variance to correct for intra-cluster correlation.

^a^*P* < 0.05 (compared to the reference group).

^b^*P* < 0.05. The standard serving size of 1 cup was defined as 50 mL in the questionnaire.

Concerning tea, the age-, sex, and baseline eGFR-adjusted revealed a non-significant higher decrease in eGFR, with β: − 0.61; 95% CI − 1.33, 0.11 mL/min/1.73 m^2^) in participants drinking ≥ 1cup tea/day compared to non-drinkers (*p*-trend = 0.10). This association was strengthened after adjustment for confounders (model 3), resulting in a significant β of − 0.93 (95% CI − 1.67, − 0.18) mL/min/1.73 m^2^ 1-year decrease in eGFR for participants drinking ≥ 1cup/day compared with non-drinkers (*p*-trend = 0.02). Similarly, for each 1 cup/day increase in tea consumption a decrease of − 0.55 (95% CI − 0.93, − 0.16) mL/min/1.73 m^2^ in eGFR (model 3) was observed (Table 2).

**Figure 1:**
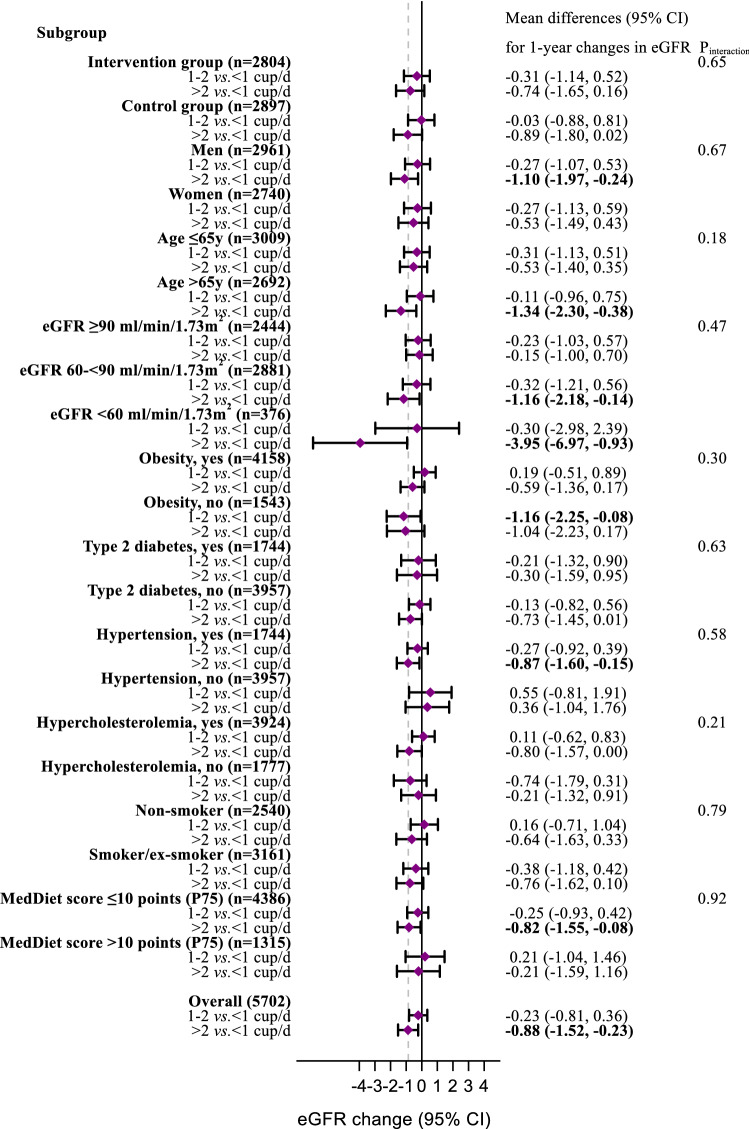
Mean multivariable-adjusted differences (95% CI) for 1-year changes in eGFR between the two highest and the lowest categories of caffeinated coffee consumption at baseline stratified by group. eGFR, estimated glomerular filtration rate; MedDiet, Mediterranean diet. Model adjusted (except the stratification factor itself) for sex (men or women), age (years, continuous), baseline eGFR (mL/min/1.73 m^2^, continuous), BMI (kg/m^2^, continuous), smoking (never, current, or former smoker), educational level (primary, secondary education or academic/graduate), physical activity (MET-min/day, continuous), type 2 diabetes prevalence (yes or no), dyslipidemia (yes or no), hypertension (yes or no), center (four categories by number of recruited participants: < 200, 200–250, 250–300, or ≥ 300), intervention group (intervention or control group), MedDiet score (17-points, continuous), total energy intake (kcal/day, quartiles), dietary intakes of total protein, saturated fat, alcohol, fiber, sodium, magnesium, potassium (each categorized at energy-adjusted quartiles), 1-year changes in systolic blood pressure (mmHg, continuous), and 1-year changes in body weight (kg, continuous), decaffeinated coffee, and tea consumption (cup/day, continuous). All analyses were conducted with robust estimates of the variance to correct for intra-cluster correlation. ^a^*P* < 0.05 (compared to the reference group). The standard serving size of 1 cup was defined as 50 mL in the questionnaire. (StataCorp. 2015. Stata Statistical Software: Release 15. College Station, TX: StataCorp LP. https://www.stata.com).

Similar results were observed when all of these analyses were repeated using percentage of change of eGFR instead of eGFR change, adjusting for baseline eGFR values (data not shown). Overall, we found no evidence that the observed associations varied across subgroups of sex, age, eGFR, obesity, diabetes, hypertension, dyslipidemia, smoking, MedDiet score at baseline, and intervention group (Fig. 1 and Supplementary Fig. S1 online).

In multivariable logistic regression models, the caffeinated coffee consumption remained significantly associated with an increased risk of a rapid eGFR decline > 3 mL/min/1.73 m^2^ when comparing > 2 versus none to < 1 cups/day (full adjusted OR, 1.19; 95% CI 1.01–1.41) (Table 3).

**Table 3:** Odds ratio (95% confidence intervals) of rapid eGFR decline (1-year changes in eGFR > 3 mL/min/1.73 m^2^) according to categories of coffee and tea consumption at baseline.

	Coffee consumption, cups/day			*P* for trend	Per cup/day increase
	0 to < 1cup/day	1–2 cups/day	> 2 cups/day		
Total coffee, n (frequency)	1091	2631	2129		
Rapid eGFR decline, cases (%)	362 (33.2)	898 (34.1)	776 (36.5)		
Model 1	1 (Ref.)	1.06 (0.91, 1.23)	1.15 (0.98, 1.35)	0.06	1.02 (0.97, 1.07)
Model 2	1 (Ref.)	1.03 (0.88, 1.20)	1.09 (0.93, 1.28)	0.24	1.00 (0.95, 1.06)
Model 3	1 (Ref.)	1.06 (0.90, 1.23)	1.11 (0.94, 1.32)	0.21	1.00 (0.94, 1.05)
Caffeinated coffee, n (frequency)	3169	1479	1203		
Rapid eGFR decline, cases (%)	1068 (33.7)	506 (34.2)	462 (38.4)		
Model 1	1 (Ref.)	1.06 (0.93, 1.21)	1.27 (1.10, 1.45)^a^	0.001	1.06 (1.01, 1.12)
Model 2	1 (Ref.)	1.05 (0.92, 1.20)	1.24 (1.07, 1.43)^a^	0.004	1.05 (1.00, 1.11)
Model 3	1 (Ref.)	1.02 (0.88, 1.18)	1.19 (1.01, 1.41)^a^	0.04	1.03 (0.97, 1.09)
Decaffeinated coffee, n (frequency)	3381	n1522	948		
Rapid eGFR decline, cases (%)	1194 (35.3)	520 (34.2)	322 (33.9)		
Model 1	1 (Ref.)	0.94 (0.82, 1.06)	0.89 (0.77, 1.04)	0.95	0.95 (0.90, 1.01)
Model 2	1 (Ref.)	0.93 (0.81, 1.06)	0.86 (0.74, 1.01)	0.05	0.94 (0.89, 1.00)
Model 3	1 (Ref.)	0.96 (0.83, 1.11)	0.87 (0.72, 1.05)	0.14	0.94 (0.87, 1.01)

	Tea consumption, cups/day			*P* for trend	Per cup/d increase
	0 cup/day	< 1cup/day	≥ 1 cups/day		
Tea, n (frequency)	3948	1243	660		
Rapid eGFR decline, cases (%)	1369 (34.7)	424 (34.1)	243 (36.8)		
Model 1	1 (Ref.)	0.97 (0.85, 1.11)	1.10 (0.92, 1.31)	0.30	1.07 (0.97, 1.17)
Model 2	1 (Ref.)	0.99 (0.87, 1.14)	1.17 (0.97, 1.40)	0.10	1.10 (1.00, 1.21)
Model 3	1 (Ref.)	0.97 (0.84, 1.12)	1.15 (0.96, 1.39)	0.12	1.10 (1.00, 1.21)

Values are means (95% CI) unless otherwise indicated.

*eGFR* estimated glomerular filtration rate, *Ref.* referent value. *P* value for trend based on multivariate logistic regression treating the median values of each category as an ordinal variable.

Model 1: adjusted for sex (men or women), age (years, continuous), and baseline eGFR (mL/min/1.73 m^2^, continuous).

Model 2: additionally adjusted for BMI (kg/m^2^, continuous), smoking (never, current, or former smoker), educational level (primary, secondary education or academic/graduate), physical activity (MET-min/day, continuous), type 2 diabetes prevalence (yes or no), dyslipidemia (yes or no), hypertension (yes or no), center (four categories by number of recruited participants: < 200, 200–250, 250–300, or ≥ 300), intervention group (intervention or control group), MedDiet score (17-points, continuous) and total energy intake (kcal/day, quartiles).

Model 3: additional adjusted for dietary intakes of total protein, saturated fat, alcohol, fiber, sodium, magnesium, potassium (each categorized by energy-adjusted quartiles), 1-year changes in systolic blood pressure (mm Hg, continuous) and 1-year changes body weight (kg, continuous). Model 3 was further adjusted (cup/day, continuous) for coffee (for tea analysis), tea (for each coffee analysis), caffeinated coffee (for decaffeinated coffee analysis), or decaffeinated coffee (for caffeinated coffee analysis). All analyses were conducted with robust estimates of the variance to correct for intra-cluster correlation.

^a^*P* < 0.05 (compared to the reference group).

^b^*P* < 0.05. The standard serving size of 1 cup was defined as 50 mL in the questionnaire.

Compared to participants in the lowest tertile of caffeine intake, in the fully adjusted model, those in the top tertile showed a significantly higher eGFR decline, with a mean difference of − 0.94 (95% CI − 1.51, − 0.38) mL/min/1.73 m^2^ (*p*-trend = 0.001) (Table 4). For each additional 50 mg/day of caffeine intake, eGFR decreased by − 0.60 (95% CI − 1.04, − 0.17) mL/min/1.73 m^2^ (model 3). When analyzed as estimated caffeine intake from coffee and tea, a similar pattern of eGFR decline was observed (Table 4). No significant association between caffeine intake from soft drinks and kidney function was observed, (tertile 3 vs. tertile 1, β: − 0.23; 95% CI − 0.82, 0.37; p-trend = 0.47).

**Table 4:** Crude and multivariate linear regression models of 1-year changes in eGFR according to tertiles of caffeine intake at baseline.

	Tertiles (T) of caffeine consumption (mg/day)			*P* for trend	Per 50 mg/day increase
	T1 (lowest) n = 1951	T2 n = 1950	T3 (highest) n = 1950		
Total caffeine (mg/day), median (P25, P75)	3.3 (0.8, 5.5)	18.8 (12.9, 22.7)	51.2 (37.7, 58.3)		
eGFR change, mL/min/1.73 m^2^	− 0.86 (− 1.28, − 0.44)	− 0.59 (− 0.99, − 0.19)	− 1.43 (− 1.82, − 1.04)		
** versus T1 (lowest)**					
Model 1	Ref	− 0.02 (− 0.57, 0.53)	− 0.89 (− 1.43, − 0.34)^a^	0.001	− 0.65 (− 1.08, − 0.23)^b^
Model 2	Ref	− 0.08 (− 0.63, 0.48)	− 0.88 (− 1.44, − 0.32)^a^	0.001	− 0.60 (− 1.03, − 0.17)^b^
Model 3	Ref	− 0.14 (− 0.68, 0.43)	− 0.94 (− 1.51, − 0.38)^a^	0.001	− 0.60 (− 1.04, − 0.17)^b^
Caffeine from coffee and tea (mg/day), median (P25–P75)	3.4 (0.8, 7.7)	16.2 (8.5, 21.6)	49.6 (28.6, 55.7)		
eGFR change, mL/min/1.73 m^2^	− 0.75 (− 1.16, − 0.33)	− 0.77 (− 1.19, − 0.36)	− 1.36 (− 1.75, − 0.98)		
**versus T1 (lowest)**					
Model 1	Ref	− 0.17 (− 0.72, 0.38)	− 0.84 (− 1.38, − 0.30)^a^	0.001	− 0.73 (− 1.23, − 0.24)^b^
Model 2	Ref	− 0.20 (− 0.78, 0.38)	− 0.86 (− 1.42, − 0.30)^a^	0.001	− 0.67 (− 1.17, − 0.17)^b^
Model 3	Ref	− 0.30 (− 0.89, 0.29)	− 0.87 (− 1.45, − 0.30)^a^	0.002	− 0.67 (− 1.19, − 0.16)^b^

Values are means (95% CI) unless otherwise indicated.

*eGFR* estimated glomerular filtration rate, *Ref.* referent value. *P* value for trend based on multivariate linear regression treating the median values of each category as an ordinal variable.

Model 1: adjusted for sex (men or women), age (years, continuous), and baseline eGFR (mL/min/1.73 m^2^, continuous).

Model 2: additionally adjusted for BMI (kg/m^2^, continuous), smoking (never, current, or former smoker), educational level (primary, secondary education or academic/graduate), physical activity (MET-min/day, continuous), type 2 diabetes prevalence (yes or no), dyslipidemia (yes or no), hypertension (yes or no), center (four categories by number of recruited participants: < 200, 200–250, 250–300, or ≥ 300), intervention group (intervention or control group), MedDiet score (17-points, continuous) and total energy intake (kcal/day, quartiles).

Model 3: additionally adjusted for dietary intakes of total protein, saturated fat, alcohol, fiber, sodium, magnesium, potassium (each categorized at energy-adjusted quartiles), 1-year changes in systolic blood pressure (mm Hg, continuous), and 1-year changes in body weight (kg, continuous). All analyses were conducted with robust estimates of the variance to correct for intra-cluster correlation.

^a^*P* < 0.05 (compared to the reference group).

^b^*P* < 0.05. Total caffeine consumption was computed from FFQs taking into account the caffeine contained in caffeinated coffee, decaffeinated coffee, tea, regular sodas, artificially sweetened soda, and chocolate.

## Discussion

In this study, we examined the association between coffee, tea, and caffeine intake and 1-year changes in eGFR in older Mediterranean individuals with overweight/obesity and MetS. We found no association between total coffee consumption and eGFR changes. However, when we explored the association by coffee subtypes, individuals who consumed > 2 cups/day of caffeinated coffee disclosed a greater eGFR decline and higher risk of rapid kidney function decline compared to those who consumed none to < 1 cup/day. Similar results were observed for drinkers of one or more cups of tea/day compared to non-consumers. Besides, participants in the highest tertile of total dietary caffeine intake showed a higher decline in eGFR than those in the reference tertile. No association of decaffeinated coffee consumption with eGFR changes was observed.

Coffee is a seed that contains a complex matrix of vitamins, minerals, bioactive phytochemicals, caffeine, diterpenes, melanoidins, and trigonelline with potential beneficial effects on human health^8,10,36^. Therefore, the association of coffee with health outcomes, especially non-communicable chronic diseases such as CKD has been of research interest in the last decade^10,11^.

Unlike our results, epidemiological studies suggest that regular coffee consumption is either unassociated with kidney function^6,7,20^ or has a protective effects against CKD in healthy middle-aged adults^21,24^. Cross-sectionally, the association between coffee and kidney function has been mainly explored in Asian populations. In two studies conducted in healthy Japanese adults^14,15^, higher eGFR was observed in usual coffee drinkers (≥ 1 cups/day) compared to non-drinkers. Similarly, in Korean women^18^, habitual coffee consumption (≥ 2 cups/day) was associated with a lower risk of CKD only in diabetic women. However, other studies in Japanese middle-aged adults reported no association between coffee and eGFR^16,17^. Notwithstanding, higher cross-sectional eGFRs values do not necessarily reflect better kidney function; therefore, these results should be taken with caution since certain kidney diseases may also manifest hyperfiltration in early stages^37^. Finally, the results of a recent meta-analysis of cross-sectional studies conducted by Wijarnpreecha et al.^7^ reported no significant association between coffee consumption and CKD risk in males, and a trend to a lower risk of CKD in women. Contrary, in our study, the associations between caffeinated coffee or tea consumption and eGFR were most apparently among men, despite interactions were not significant. Further research is warranted to clarify the differences observed by sex, and if they are proven to understand the plausible mechanisms involved.

Recently, prospective studies have reported inconsistent results. Three studies, one conducted in US adults^21^ and two in Asian populations^19,24^, reported a decreased risk of incident CKD in healthy individuals with greater coffee consumption. In contrast, a study conducted in an Iranian population revealed no association between coffee and CKD incidence after 6 years of follow-up^22^. The only epidemiological study conducted in an European population reported a cross-sectional positive association between coffee consumption and eGFR at baseline, but no prospective association with eGFR changes after 15 year follow-up^20^. Until now, the only RCT in 19 young Japanese revealed a beneficial effect of coffee consumption for 2 weeks, but not green tea, on cystatin-C-based eGFR^23^.

The main discrepancies between previous studies and our observations might be attributable to the populations examined, follow-up time, and the assessment tools used. Most studies were conducted in apparently healthy middle-aged populations, and none of them included overweight/obese older adults at high cardiovascular risk. Additionally, previous studies have focused on exploring the association between total coffee and kidney function, but none of them have explored differences by coffee subtypes. Further, we also determined whether the observed detrimental effects of caffeinated coffee and tea consumption on kidney function differed across subgroups according to various baseline factors and similar trends were seen; this type of analysis was seldom performed in previous studies.

The association between tea and kidney dysfunction has been less explored and the few studies in the field had reported no significant associations^15,19,20,22^. We observed that the consumption of ≥ 1cup tea/day was associated with higher eGFR decline. Despite, in our study tea consumption was lower than caffeinated coffee consumption, our findings regarding caffeine intake suggest that this nutrient could be the causal for the deleterious renal effect observed.

Additionally, we found no association between decaffeinated coffee and kidney function, that might reinforce the potentially harmful effect of caffeine^13^. However, the mechanism that confers this negative effect of caffeine intake is uncertain^36^. A possible explanation could be related with the structural similarity of caffeine and adenosine acting as a nonselective antagonist of A1 adenosine receptors, which may be a potential mechanism for the impairment of renal function^11^. On distal afferent arterioles, A1 adenosine receptor activation causes vasoconstriction and decreases eGFR^38^. By contrast, adenosine antagonism by caffeine may prevent afferent arteriolar constriction or induce vasodilation, increasing renal plasma flow and acute elevation of eGFR^11^. Probably this increase in eGFR could lead to acute compensatory hyperfiltration and subsequent accelerated decline in GFR^37^. This hypothesis may be partly supported by the observed relationship in our study between caffeinated coffee and a slightly higher baseline eGFR and, subsequently, a higher 1-year eGFR decline. In this context, it has been reported that, compared to decaffeinated coffee, caffeinated coffee could cause a short-lasting increase in blood pressure in healthy young subjects^39^. Also, in young-to-middle-age hypertensive adults, caffeinated coffee drinking has been linked with glomerular hyperfiltration^40^, which may be exaggerated in older individuals with pre-existing comorbidities^13^ (e.g., hypertension) as our studied population, but warrants further studies. In fact, in experimental studies conducted in obese diabetic rats, administration of caffeine for 2 weeks induced early renal injury characterized by proteinuria, increased renal vascular resistance, and increased heart rate^41^. Moreover, the same authors reported that long-term caffeine consumption exacerbated renal failure and induced more severe tubule-interstitial and glomerular damage^42^. Nevertheless, this has not been explored in humans and RCTs are needed.

Our study has some strengths. First, the relatively large sample size, its prospective design, and adjustment for many potential confounding factors. Secondly, we examined the association between eGFR and coffee subtypes, which gives greater insight into the potential effect of coffee and caffeine on kidney function. Finally, we conducted sub-group analyses to examine whether kidney function decline was accelerated by unhealthy behaviors (e.g., smoking or a poor diet) or pre-existing comorbid conditions. Nevertheless, we also acknowledge some limitations. It should be underlined that the caffeine content in coffee, tea, and other beverages (e.g., soft drinks) and foods varies greatly, which may lead to misclassification, and also that coffee composition can depend on the type of coffee bean and the brewing process used, which may influence the biological effects it has on the human organism. Also, SCr-based eGFR, a common biomarker of kidney function used in almost all epidemiologic studies, may overestimate true GFR values. Finally, our study was conducted in aged Mediterranean individuals with MetS, therefore our findings cannot be extrapolated to other populations.

In summary, caffeinated coffee and tea consumption and caffeine intake were associated with a greater 1-year decline of renal function in older individuals at high cardiovascular risk. Further studies are needed to clarify these associations; if proven, advice on coffee and tea consumption along with caffeine intake should be included in nutritional strategies for kidney disease prevention, particularly in individuals at high risk.

## Supplementary Information

Supplementary Figure.

## Data Availability

The dataset (including data dictionaries) of PREDIMED-Plus is available to external investigators in order to make possible the replication of the main analyses used for the published article. However, due to the restrictions imposed by the Informed Consent and the Institutional Review Boards (IRB), bona fide investigators interested in analyzing the PREDIMED-Plus dataset may submit a brief proposal and statistical analysis plan to the corresponding author (JS-S) at jordi.salas@urv.cat. Upon approval from the Steering Committee and IRBs, the data will be made available to them using an onsite secure access data enclave. The study protocol is available at http://predimedplus.com/.

## Abbreviations

ARBs: Angiotensin-type 2 receptor blockers
ACEis: Angiotensin converting enzyme inhibitors
CKD: Chronic Kidney Disease
CKD-EPI: Chronic Kidney Disease Epidemiology Collaboration
CIs: Confidence intervals
DBP: Diastolic blood pressure
eGFR: Estimated glomerular filtration rate
HDL-c: High-density lipoprotein-cholesterol
MedDiet: Mediterranean diet
MetS: Metabolic syndrome
BMI: Body mass index
PREDIMED: PREvención con DIeta MEDiterranea
SFFQ: Semiquantitative food-frequency questionnaire
SBP: Systolic blood pressure

## References

[1] Xie Y, Bowe B, Mokdad AH, Xian H, Yan Y, Li T (2018). Analysis of the Global Burden of Disease study highlights the global, regional, and national trends of chronic kidney disease epidemiology from 1990 to 2016. Kidney Int..

[2] Mallappallil M, Friedman EA, Delano BG, McFarlane SI, Salifu MO (2014). Chronic kidney disease in the elderly: evaluation and management. Clin Pract..

[3] Gansevoort RT, Correa-Rotter R, Hemmelgarn BR, Jafar TH, Heerspink HJL, Mann JF (2013). Chronic kidney disease and cardiovascular risk: epidemiology, mechanisms, and prevention. Lancet.

[4] Campbell KL, Carrero JJ (2016). Diet for the management of patients with chronic kidney disease; it is not the quantity, but the quality that matters. J. Ren. Nutr..

[5] Goraya N, Wesson DE (2015). Dietary interventions to improve outcomes in chronic kidney disease. Curr. Opin. Nephrol. Hypertens..

[6] Kennedy OJ, Roderick P, Poole R, Parkes J (2017). Coffee and kidney disease. Int. J. Clin. Pract..

[7] Wijarnpreecha K, Thongprayoon C, Thamcharoen N, Panjawatanan P, Cheungpasitporn W (2017). Association of coffee consumption and chronic kidney disease: a meta-analysis. Int. J. Clin. Pract..

[8] Ludwig IA, Clifford MN, Lean MEJ, Ashihara H, Crozier A (2014). Coffee: biochemistry and potential impact on health. Food Funct..

[9] McKay DL, Blumberg JB (2002). The role of tea in human health: an update. J. Am. Coll. Nutr..

[10] Poole R, Kennedy OJ, Roderick P, Fallowfield JA, Hayes PC, Parkes J (2017). Coffee consumption and health: umbrella review of meta-analyses of multiple health outcomes. BMJ.

[11] Higdon JV, Frei B (2006). Coffee and health: a review of recent human research. Crit. Rev. Food Sci. Nutr..

[12] Di Lorenzo A, Curti V, Tenore GC, Nabavi SM, Daglia M (2017). Effects of tea and coffee consumption on cardiovascular diseases and relative risk factors: an update. Curr. Pharm. Des..

[13] Lim D, Chang J, Ahn J, Kim J (2020). Conflicting effects of coffee consumption on cardiovascular diseases: does coffee consumption aggravate pre-existing risk factors?. Processes.

[14] Kotani K, Sakane N, Yamada T, Taniguchi N (2010). Association between coffee consumption and the estimated glomerular filtration rate in the general Japanese population: preliminary data regarding C-reactive protein concentrations. Clin. Chem. Lab. Med..

[15] Nakajima K, Hirose K, Ebata M, Morita K, Munakata H (2010). Association between habitual coffee consumption and normal or increased estimated glomerular filtration rate in apparently healthy adults. Br. J. Nutr..

[16] Pham NM, Yoshida D, Morita M, Yin G, Toyomura K, Ohnaka K (2010). The relation of coffee consumption to serum uric acid in Japanese men and women aged 49–76 years. J. Nutr. Metab..

[17] Miyatake N, Shikata K, Makino H, Numata T, Miyatake N, Shikata K (2011). The relation between estimated glomerular filtration rate (eGFR) and coffee consumption in the Japanese. Health (Irvine, CA).

[18] Kim BH, Park YS, Noh HM, Sung JS, Lee JK (2013). Association between coffee consumption and renal impairment in Korean women with and without diabetes: analysis of the Fourth Korea National Health and Nutrition Examination Survey in 2008. Korean J. Fam. Med..

[19] Lew Q-LJ, Jafar TH, Jin A, Yuan J-M, Koh W-P (2018). Consumption of coffee but not of other caffeine-containing beverages reduces the risk of end-stage renal disease in the Singapore Chinese Health Study. J. Nutr..

[20] Herber-Gast G-CM, van Essen H, Verschuren WM, DA Stehouwer C, Gansevoort RT, Bakker SJ (2016). Coffee and tea consumption in relation to estimated glomerular filtration rate: results from the population-based longitudinal Doetinchem Cohort Study. Am. J. Clin. Nutr..

[21] Hu EA, Selvin E, Grams ME, Steffen LM, Coresh J, Rebholz CM (2018). Coffee consumption and incident kidney disease: results from the atherosclerosis risk in communities (ARIC) study. Am. J. Kidney Dis..

[22] Gaeini Z, Bahadoran Z, Mirmiran P, Azizi F (2019). Tea, coffee, caffeine intake and the risk of cardio-metabolic outcomes: findings from a population with low coffee and high tea consumption. Nutr. Metab. (Lond.).

[23] Saito M, Nemoto T, Tobimatsu S, Ebata M, Le Y, Nakajima K (2011). Coffee consumption and cystatin-C-based estimated glomerular filtration rates in healthy young adults: results of a clinical trial. J. Nutr. Metab..

[24] Jhee JH, Nam KH, An SY, Cha M-U, Lee M, Park S (2018). Effects of coffee intake on incident chronic kidney disease: a community-based prospective cohort study. Am. J. Med..

[25] Alberti KGMM, Eckel RH, Grundy SM, Zimmet PZ, Cleeman JI, Donato KA (2009). Harmonizing the metabolic syndrome: a joint interim statement of the International Diabetes Federation Task Force on Epidemiology and Prevention; National Heart, Lung, and Blood Institute; American Heart Association; World Heart Federation; International. Circulation.

[26] Martínez-González MA, Buil-Cosiales P, Corella D, Bulló M, Fitó M, Vioque J (2019). Cohort profile: design and methods of the PREDIMED-Plus randomized trial. Int. J. Epidemiol..

[27] Salas-Salvadó J, Díaz-López A, Ruiz-Canela M, Basora J, Fitó M, Corella D (2019). Effect of a lifestyle intervention program with energy-restricted Mediterranean diet and exercise on weight loss and cardiovascular risk factors: one-year results of the PREDIMED-Plus trial. Diabetes Care.

[28] Fernández-Ballart JD, Piñol JL, Zazpe I, Corella D, Carrasco P, Toledo E (2010). Relative validity of a semi-quantitative food-frequency questionnaire in an elderly Mediterranean population of Spain. Br. J. Nutr..

[29] Moreiras O, Carbajal A, Cabrera L, Cuadrado C (2018). Tablas de composición de alimentos: guía de prácticas [food composition tables: practical guides].

[30] Zucconi S, Volpato C, Adinolfi F, Gandini E, Gentile E, Loi A (2013). Gathering consumption data on specific consumer groups of energy drinks. EFSA Support Publ..

[31] Levey AS, Stevens LA, Schmid CH, Zhang YL, Castro AF, Feldman HI (2009). A new equation to estimate glomerular filtration rate. Ann. Intern. Med..

[32] Molina L, Sarmiento M, Peñafiel J, Donaire D, Garcia-Aymerich J, Gomez M (2017). Validation of the Regicor short physical activity questionnaire for the adult population. Lucía A, editor. PLoS ONE.

[33] Martínez-González MA, López-Fontana C, Varo JJ, Sánchez-Villegas A, Martinez JA (2005). Validation of the Spanish version of the physical activity questionnaire used in the Nurses’ Health Study and the Health Professionals’ Follow-up Study. Public Health Nutr..

[34] Schröder H, Fitó M, Estruch R, Martínez-González MA, Corella D, Salas-Salvadó J (2011). A short screener is valid for assessing Mediterranean diet adherence among older Spanish men and women. J. Nutr..

[35] Willett W (1998). Nutritional Epidemiology.

[36] Godos J, Pluchinotta FR, Marventano S, Buscemi S, Li Volti G, Galvano F (2014). Coffee components and cardiovascular risk: beneficial and detrimental effects. Int. J. Food Sci. Nutr..

[37] Tuttle KR (2017). Back to the future: glomerular hyperfiltration and the diabetic kidney. Diabetes.

[38] Vallon V, Osswald H (2009). Adenosine receptors and the kidney. Handb. Exp. Pharmacol..

[39] Mahmud A, Feely J (2001). Acute effect of caffeine on arterial stiffness and aortic pressure waveform. Hypertens (Dallas, Tex 1979).

[40] Palatini P, Dorigatti F, Saladini F, Benetti E, Mos L, Mazzer A (2012). Factors associated with glomerular hyperfiltration in the early stage of hypertension. Am. J. Hypertens..

[41] Tofovic SP, Salah EM, Jackson EK, Melhem M (2007). Early renal injury induced by caffeine consumption in obese, diabetic ZSF1 rats. Ren. Fail..

[42] Tofovic SP, Kost CK, Jackson EK, Bastacky SI (2002). Long-term caffeine consumption exacerbates renal failure in obese, diabetic, ZSF1 (fa-fa(cp)) rats. Kidney Int..

