# Supplementary figures and images for: Consumption of caffeinated beverages and kidney function decline in an elderly Mediterranean population with metabolic syndrome
Source: Sci Rep. 2021 Apr 22;11:8719. doi: 10.1038/s41598-021-88028-7 (PMC8062443; doi:10.1038/s41598-021-88028-7)

## Slide 1
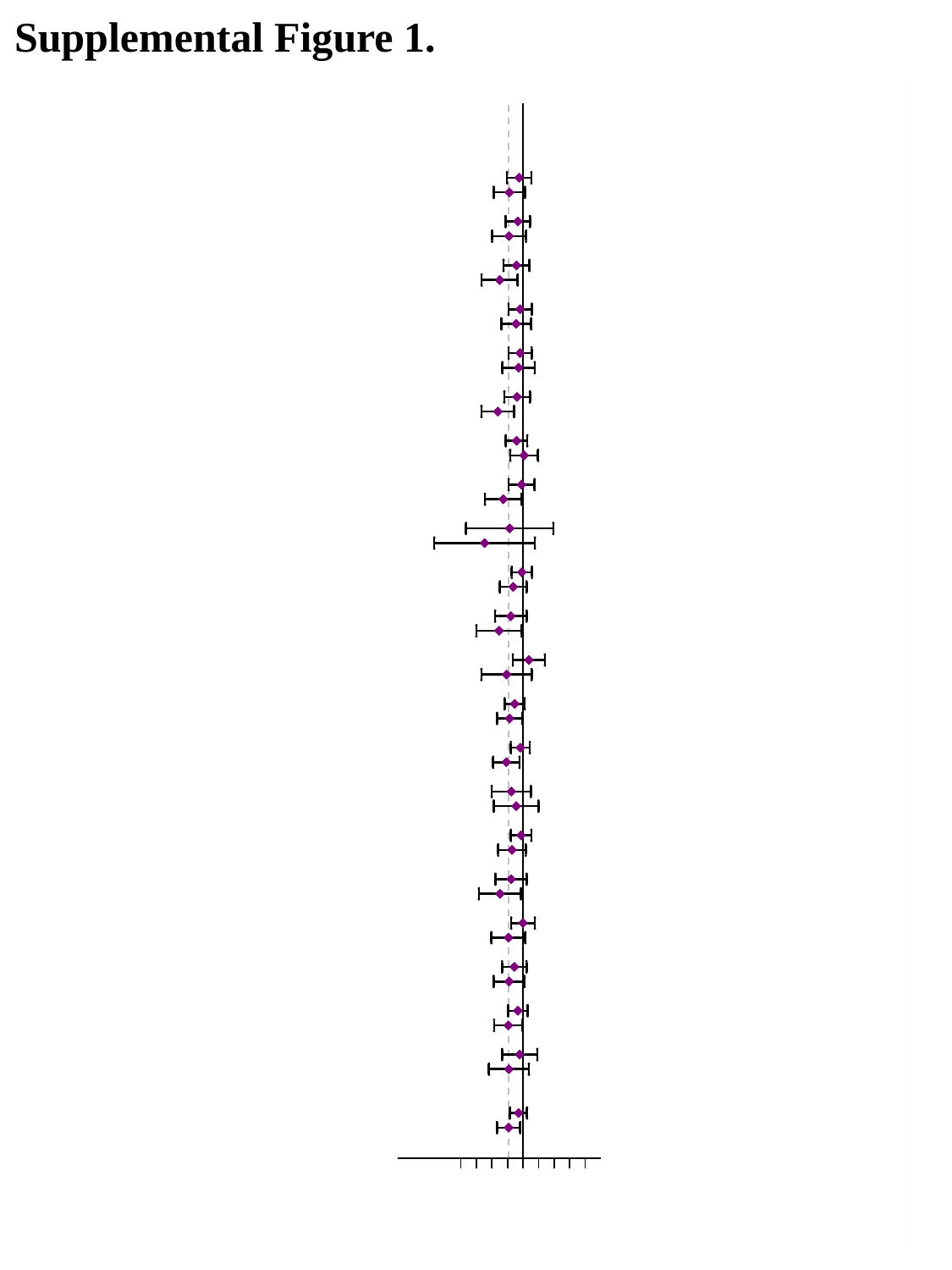

Supplemental Figure 1.

Supplement: Supplementary file 1 — Supplementary Figure. [file 41598_2021_88028_MOESM1_ESM.pptx]
